# Commonalities in the Features of Cancer and Chronic Fatigue Syndrome (CFS): Evidence for Stress-Induced Phenotype Instability?

**DOI:** 10.3390/ijms23020691

**Published:** 2022-01-08

**Authors:** Andrej Rusin, Colin Seymour, Alan Cocchetto, Carmel Mothersill

**Affiliations:** 1 Department of Integrated Biomedical Engineering and Health Sciences, McMaster University, Hamilton, ON L8S 4L8, Canada; 2 Department of Biology, McMaster University, Hamilton, ON L8S 4L8, Canada; seymouc@mcmaster.ca (C.S.); mothers@mcmaster.ca (C.M.); 3 National CFIDS Foundation Inc., 103 Aletha Road, Needham, MA 02492, USA; acocchetto@gmail.com

**Keywords:** chronic fatigue and immune dysfunction syndrome (CFIDS), myalgic encephalomyelitis/chronic fatigue syndrome (CFS/ME), cancer, radiation, mitochondria, non-targeted effects (NTE), metabolism, biomarkers

## Abstract

Chronic Fatigue Syndrome/Myalgic Encephalomyelitis (CFS/ME) and Cancer-Related Fatigue (CRF) are syndromes with considerable overlap with respect to symptoms. There have been many studies that have compared the two conditions, and some of this research suggests that the etiologies of the conditions are linked in some cases. In this narrative review, CFS/ME and cancer are introduced, along with their known and putative mechanistic connections to multiple stressors including ionizing radiation. Next, we summarize findings from the literature that suggest the involvement of HPA-axis dysfunction, the serotonergic system, cytokines and inflammation, metabolic insufficiency and mitochondrial dysfunction, and genetic changes in CRF and CFS/ME. We further suspect that the manifestation of fatigue in both diseases and its causes could indicate that CRF and CFS/ME lie on a continuum of potential biological effects which occur in response to stress. The response to this stress likely varies depending on predisposing factors such as genetic background. Finally, future research ideas are suggested with a focus on determining if common biomarkers exist in CFS/ME patients and those afflicted with CRF. Both CFS/ME and CRF are relatively heterogenous syndromes, however, it is our hope that this review assists in future research attempting to elucidate the commonalities between CRF and CFS/ME.

## 1. Introduction to Chronic Fatigue Syndrome/Myalgic Encephalomyelitis (CFS/ME)

Chronic Fatigue Syndrome/Myalgic Encephalomyelitis (CFS/ME) is a variably severe disease that presents clinically as a multi-symptom, recurring illness [1]. Patients commonly report a panel of symptoms, with persistent fatigue being the most indicative diagnostic sign [2,3,4]. While the disease is heterogenous in nature and diverse biological effects appear differently between patients, the underlying syndrome is generally hypothesized to be the result of some combination of cognitive, immune, and endocrine dysfunction [5,6,7]. Common symptoms include sleep disturbances, orthostatic intolerance, chronic pain, challenges to memory and cognition (or “brain fog”), weakness, general malaise, tender lymph nodes, digestive problems including irritable bowel syndrome, night sweats and chills, allergies, irregular heartbeat, shortness of breath, and sore throat [8,9,10,11,12,13]. The onset of CFS/ME has been observed to manifest following exposure to an acute stressor in many patients, such as a viral or bacterial infection [12,14]. The onset of the syndrome can be gradual or sudden. CFS/ME patients often report exacerbation of their symptoms following relatively minor physical or mental activity; this is known as post-exertional malaise (PEM) [15]. This oftentimes results in the complete inability—or significantly reduced capacity—of those suffering from the disease to perform routine tasks, consequently diminishing performance at school and work. CFS/ME appears more frequently in those of European heritage but is not believed to be necessarily more common in this population. CFS/ME is believed to affect millions internationally with varying degrees of medical recognition [8,16,17,18,19]. The disease is significantly more common in women compared to men, and usually affects those between 40 and 60 years of age. However, studies suggest that the disease can develop in children as well, with greater frequency in adolescents than younger children [20]. The cause of this distribution is unknown; however, it may suggest that aging or repeated exposure to inciting factors over time can cause or contribute to the appearance or progression of the disease.

Currently, a diagnosis of CFS/ME is always made based on symptomatology, as no confirmed biomarker or diagnostic test exists [3,21]. The Fukuda and the US Center for Disease Control and Prevention (CDC) criteria are most frequently used to make diagnoses of CFS/ME based on symptoms related to fatigue, PEM, cognitive dysfunction, and sleep disturbances [22,23]. Other diagnostic criteria exist, including the Institute of Medicine (IOM) and U.S. CFS/ME Expert Physician Coalition criteria. While it is estimated that the disease affects millions internationally, physician skepticism of the disease and consequent dismissal of patients was and continues to be a significant obstacle for diagnosis [24,25,26,27]. A survey of approximately 800 general physicians in the United Kingdom revealed that 48% did not feel confident diagnosing the disease and 41% did not feel confident in the available treatments [28]. Another survey of physicians in the United States from 2010 found that the majority (80%) could correctly identify CFS/ME symptoms, while 40% had given a diagnosis of CFS/ME at some point in their medical practice; this indicates that attitudes towards the illness in healthcare may be changing, with increased awareness and greatly reduced negative attitudes compared to previous years [29]. However, it is known that CFS/ME patients are medically underserved in the United States with regard to specialist care access; the majority cited geographic and financial barriers that precluded access to medical specialists, even though nearly all participants expressed interest in such care [30]. 

Often, it is very difficult to separately assess the metabolic, immunologic, and neurologic manifestations of the disease [31,32]. Usually, effects falling into one or more of these categories influence processes and outcomes in other categories, which is discussed in further detail in later sections of this review. Among epidemiological studies, it is difficult to compare reports because there is no universal set of criteria used to diagnose CFS/ME [5] and the biomedical assays performed that may be indicative of CFS/ME can vary greatly from study to study. These factors significantly complicate the investigation, diagnosis, and treatment of CFS/ME. In this narrative review, we hypothesize that CFS/ME could be connected to cancer-related fatigue.

### CFS/ME, Ionizing Radiation, and Multiple Stressors

It is also of note that similar syndromes have been reported in other groups exposed to acute stressors, like Atomic and Gulf War Veterans, radiotherapy patients and cancer survivors, and survivors of nuclear catastrophes like the one that occurred in Chernobyl [33,34,35,36]. This may suggest that the underlying pathophysiology of CFS/ME and syndromes associated with exposure to ionizing radiation may share some commonalities. Furthermore, it may suggest that ionizing radiation exposure can cause CFS/ME [33]; however, this has not been conclusively demonstrated.

The first paper that proposed that CFS/ME may result following exposure to ionizing radiation studied Chernobyl liquidators following the disaster [33]. In the aftermath of the 1986 nuclear disaster in the Soviet Union that precipitated the uncontrolled release of over one hundred types of radioisotopes into the environment, teams made up of civilians, police, military, and firefighters, were tasked with organizing and executing clean-up efforts. This action was undertaken to mitigate further uncontrolled release and subsequent contamination. A subset of this group, containing liquidators which were estimated to have been exposed to less than 300 mSv, was studied for health effects. In a sample of 100 workers, 26 fit the diagnostic criteria for CFS/ME, with the authors noting persistent fatigue, malaise, and immune dysfunction. This was initially suspected to be the result of neurological damage (vegetative-vascular dystonia) linked to low-dose radiation exposure [37], which would otherwise be considered a subclinical effect of exposure in this context due to its presentation. Another study by the same group sought to identify biological markers associated with radiation exposure in Chernobyl liquidators [38]. In this later study, the authors found several markers associated with functional neurological dysfunction, which was proposed to be a cause of the symptoms. It was also observed that the prevalence of CFS/ME in Chernobyl liquidators decreased significantly after 10 years. Together, these reports described that the extent of neurodegeneration associated with participating in the liquidation was correlated with estimated dose as well as time spent in high radiation areas, indicating a direct connection between CFS/ME symptoms and the extent of radiation exposure.

It should be noted that other survivors of radiation accidents, Atomic and Gulf War veterans, and radiotherapy patients have experienced similar symptoms following ionizing radiation exposure [13,34,39,40,41,42,43,44,45,46]. A clear dose-dependence was not always observed in the literature—consistent with low dose radiation effects such as radiation-induced bystander effects—but suggestions that ionizing radiation exposure may be linked to fatigue have been historically dismissed as unfounded. This may be due, in part, to the lack of clear dose-dependence, the greater prevalence of deterministic effects with higher doses, and assumptions of radiophobia in those reporting symptoms [26,47,48,49,50]. There are other characteristics of non-targeted effects that make them a candidate mechanism for explaining CFS/ME in some cases [35]. Non-targeted effects are most prevalent following exposure to lower doses of ionizing radiation (below 0.5 Gy) and they saturate at higher doses [51,52,53,54]. These effects are also known to persist over time and across generations due to their induction of genomic instability and additional signaling following receipt of a primary signal [55,56,57]. It is therefore proposed that non-targeted effects like radiation-induced bystander effects could promote the inflammatory responses [58,59,60] seen in CFS/ME patients and contribute in yet undiscovered ways to promoting fatigue and related symptoms. This could occur by modulation of oxidative metabolism for example, which has been observed in several studies of a subset of non-targeted effects known as radiation-induced bystander effects (RIBE) [61,62]. This is discussed in further detail later in this review.

**Figure 1:**
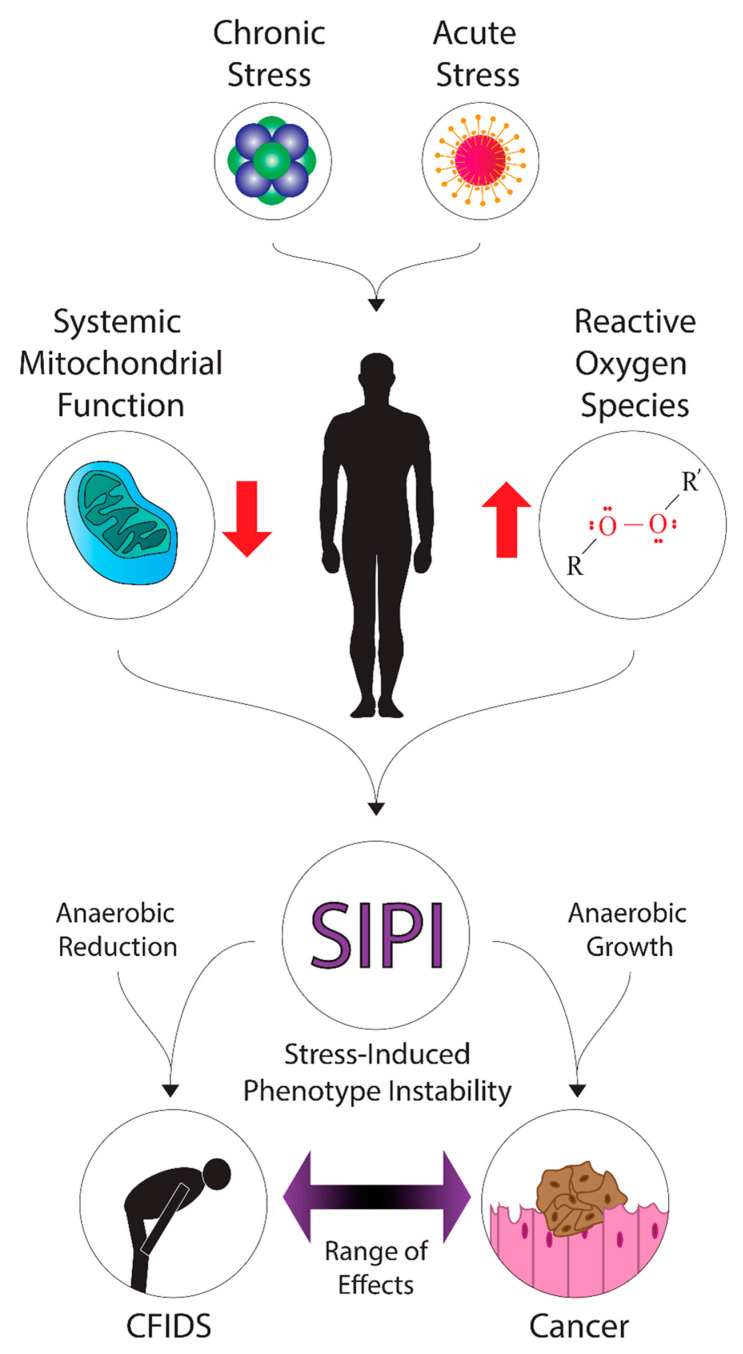
A diagram of the hypothetical mechanisms and effects of stress-induced phenotype instability in cancer and CFS/ME. Various forms of acute stress (such as radiation exposure or infection) or chronic stress may contribute to the systemic dysregulation of energy metabolism and upregulation of oxidative stress. These factors may promote stress-induced phenotype instability in patients, where down- or up-regulation of metabolic pathways could contribute to energy deficits or growth, leading to fatigue symptoms in cancer and CFIDS patients.

Furthermore, we suspect that CFS/ME may be connected mechanistically to cancer and believe that fatigue as a common symptom may indicate this connection. To understand why this is a possibility, a review of the fundamentals of cancer biology is required. Here, we will review the distinctive features of cancer, what biological changes are associated with cancer, and how cancer is linked to multiple stressors and ionizing radiation exposure. A pictorial summary of our hypotheses is presented in Figure 1.

## 2. Proposed Link between CFS/ME and Cancer

We suspect that CFS/ME and cancer are linked. Some authors have proposed a link between CFS/ME and cancer, although this link has yet to be conclusively demonstrated. One study noted that fatigue symptoms in CFS/ME patients and those suffering from cancer-related fatigue (CRF) show some similarities [63]. 

Most cancers arise spontaneously and may be associated with genetic predisposition and environmental factors [64,65,66]. Environmental causes include viral and bacterial infection [67,68,69,70,71,72], smoking [73,74], diet, and lifestyle choices such as sedentation [75,76]. Certain syndromes are known to predispose individuals to specific types of cancer, and these are typically inherited. Comparatively, a few authors proposed that CFS/ME may have a basis, or is at least “buffered”, by genetics. 

Tumors modulate their metabolism to allow for further growth and invasion of surrounding tissues. It is now known that cancer cells perform fermentation after glycolysis over oxidative phosphorylation because transformed cells have different nutritional demands than normal cells. Tumor cells shunt pyruvate to a fermentative pathway because this is conducive to the accumulation of biomass required for growth and division, even though catabolism and extensive oxidation to carbon dioxide produces more energy in the form of ATP [77,78,79]. The metabolites generated in glycolysis can be utilized to synthesize a diverse number of biomolecules and can be used in the pentose phosphate pathway (glucose-6-phosphate) [80,81]. Glycogen synthesis also starts with metabolites from glycolysis (glucose-6-phosphate) [82,83], as does the synthesis of glycerol (glyceraldehyde-3-phosphate), fatty acid synthesis (pyruvate) [84,85], and cholesterol synthesis (pyruvate). Further, the metabolites of the citric acid cycle—which is supplied by the products of glycolysis and the dehydrogenation of pyruvate—can be used for nucleic and amino acid synthesis [86]. It is suspected that the modulation of cellular metabolism is also at play in CFS/ME; this is further described in Section 3.5. 

### Connections to Multiple Stressors

As previously discussed, carcinogenesis can usually be linked to some combination of genetic or environmental factors. This may also be the case for CFS/ME, and this is discussed in further detail in later sections. Certain inherited conditions can predispose individuals to cancer formation [87,88]. Specific mutations in certain genes can greatly predispose an individual to oncogenesis, like mutations in the tumor suppressors BRCA1 and BRCA2, which greatly increase the risk of developing breast and ovarian cancers [66,89,90].

Furthermore, a variety of environmental factors are important in the induction of tumorigenesis. It is now widely recognized that smoking significantly increases one’s risk of developing many different types of cancer, from cancers of the upper and lower respiratory tract to cancers of the bladder, kidney, pancreas, and stomach [91,92,93]. It is known that chemical carcinogens present in cigarette smoke promote DNA damage and allow the accumulation of mutations in tumor suppressors and oncogenes. Moreover, radioactive carcinogens are also present in cigarette smoke that contribute to oncogenesis. Finally, the liberation of free radicals and pro-oxidant chemicals in cigarette smoke damages blood vessels and promotes oncogenesis through changes in the tissue microenvironment, causing inflammation and damage to structures like bronchi and alveoli [91,92,93]. Other environmental causes are known to promote tumorigenesis. For example, microparticle and nanoparticle exposure is an occupational hazard for some, including those in the mining industry. Furthermore, naturally-occurring radon is another hazard that is prevalent in many parts of the world [94]. Viral and bacterial infection is another cause of some types of cancer. For example, *Helicobacter pylori* can cause gastric cancers via chronic inflammation of stomach mucosa and the secretion of carcinogenic virulence factors [68,69]. Chronic inflammation can lead to DNA damage over time, which can cause random alterations to DNA if not repaired. The same link to environmental toxins and stressors has not been conclusively demonstrated in CFS/ME yet, although some evidence is present in the literature [95,96].

#### Cancer and Radiation Damage

Radiation exposure has been widely observed as a risk factor for oncogenesis for many years. Ionizing radiation damages DNA and produces double-strand breaks; these breaks, if not repaired, lead to mutations in the cell and its progeny [97]. If the error is in an essential gene, or if there is an accumulation of many mutations, death of the cell may occur. This could also damage genetic information that, in turn, leads to the manifestation of CFS/ME through several mechanisms, which are described in further detail in Section 3. Alternatively, a cell may become transformed if the mutations occur in tumor suppressors or proto-oncogenes [98,99]. Although this direct damage is believed to be the primary cause of mutations resulting in oncogenesis, other radiation interactions promote DNA damage or a microenvironment conducive to transformation. These and other forms of indirect damage are collectively referred to as the non-targeted effects of ionizing radiation (non-targeted effects) [100,101], which were discussed at some length earlier in this review. These are known as “non-targeted” because the damage occurs in cells that have not received a direct deposition of energy and are therefore outside of the field of radiation classically associated with damage to biomolecules [97]. It is also possible that non-targeted effects could account for the etiology of CFS/ME in some cases, and could also be the reason why a dose-dependent relationship between radiation exposure and CFS/ME has not yet been identified [34]. 

The term non-targeted effects encompasses a broad range of effects in cells and tissues in vitro and in vivo. RIBE were demonstrated in vitro and named as such by a few groups in the early and late nineties; however, the evidence for radiation-induced bystander effects appears to be almost as old as the discovery of radioactivity [102,103,104]. Next, a demonstration of bystander signals as soluble factors secreted by exposed cells into a culture medium was performed by Mothersill and Seymour [53]. Shortly thereafter, a great number of reports were published indicating roles for mitochondria, reactive oxygen species, and metabolism in the manifestation of bystander effects in recent decades [61,105,106,107]. 

As previously discussed, tumor cells typically alter cellular metabolism such that metabolites are directed to anabolic pathways, while catabolic pathways, such as the energy-generating reactions in the citric acid cycle and mitochondrial oxidative phosphorylation, are down-regulated [108,109,110]. RIBE are known to modulate aerobic metabolism in vitro, specifically acting on mitochondria and repressing mitochondrial respiratory chain activity [61,62]. Additionally, a number of mitochondrial changes are implicated in bystander signaling, including changes in morphology, mutations in mitochondrial DNA, and loss of the inner mitochondrial membrane potential required to drive ATP synthesis [61,107,111,112,113]. The result of this signaling is the loss of ATP production [77], generation of oxidative stress [114], and ultimately apoptosis [107,115]. Apoptosis can be initiated through a signaling pathway specific to mitochondria; proapoptotic members of the BCL-2 family facilitate the permeabilization of the mitochondrial membrane [115,116] which is usually coincident with the leakage of electrons from enzymes in the electron transport chain [117]. Cytochrome c, the terminal electron carrier in the transport chain, is liberated from the mitochondria and activates apoptotic enzymes [118]. As discussed below, all of these changes to mitochondrial and aerobic metabolism could potentially be implicated in CFS/ME as well, although further research is still required to conclusively demonstrate mitochondrial dysfunction or biomarkers. 

## 3. Do Cancer and CFS/ME Share a Common Etiology?

There are a few common features that may connect cancer to CFS/ME. In Supplementary Table S1, we summarize the findings of each article that was reviewed in the following section. There are similarities and differences in the type of fatigue experienced by CFS/ME patients and cancer patients. CFS/ME patients experience post-exertional malaise (PEM), which is defined as the worsening of symptoms upon minimal physical or mental exertion [119]. In contrast to CFS/ME-related fatigue, cancer-related fatigue is not typically associated with post-exertional malaise [120]. This difference could point to differences in the mechanisms underlying the diseases, or potentially a differential systemic response to external stressors. For some time, researchers have questioned whether CFS/ME or similar syndromes could predispose individuals to or indicate carcinogenesis. Since the advent of cancer therapy, cancer-related fatigue is a syndrome that has been observed in many neoplastic diseases and affects most of those with cancer [121]. This fatigue is often associated with symptomatology that mirrors CFS/ME, including other symptoms like chronic pain, sleep disturbances, cognitive dysfunction, and emotional distress. This fatigue and related symptoms may be associated with the burden of radiation and/or chemotherapy treatment on the body. However, fatigue is also common in those not undergoing treatment, when a cancer is in remission, and even prior to diagnosis. The pathophysiology of cancer-related fatigue is poorly understood, but sometimes it is possible to determine whether the fatigue is related to the treatment or disease alone; if fatigue symptoms are exacerbated following treatment but subside upon suspension of the regime, then it may be concluded that the fatigue results from treatment [122,123,124]. As stated previously, both chemotherapy and radiation therapy are known to induce fatigue in patients, and the onset and severity of fatigue typically follow a reproducible course if treatment is suspended and resumed at a later period [43,46,125]. There is also evidence that changes induced by cancer, such as increased energy and metabolite demand, may be responsible for cancer-related fatigue. Some proposed mechanisms that may underpin CRF include inflammation and induction of cytokine signaling, disruption of sleep cycles and the circadian rhythm, disruption of the hypothalamic-pituitary-adrenal (HPA) axis, muscle loss, and nutritional deficit [122]. One systematic review of the literature found that cancer-related fatigue was consistently linked to immune and inflammatory responses, metabolic changes, neuroendocrine changes, and changes in genetic biomarkers [123]. Idiopathic fatigue can be distinguished from secondary fatigue caused by cancer-induced changes; for example, fatigue can also be a symptom indicative of anemia, and leukemias may cause anemia due to the destruction of bone marrow [126]. If the underlying problem can be addressed, such as nutritional deficiencies or side effects from medications, then the fatigue symptom can likely be effectively treated.

Even though CRF and CFS/ME share some common features, along with differences, the connection between the two has not been conclusively established. While there are reports that attempt to refute any relationship between the syndromes, there are several studies that investigate whether CRF and CFS/ME are coincident and etiologically linked. Based on one reviewed study, there is a general increased risk of earlier mortality in CFS/ME patients compared to the general US population. Interestingly, CFS/ME patients showed a lower age of death for suicide and cancer (cancer: M = 66.3 years; 71.1 years), indicating that CFS/ME patients with cancer tended towards poorer outcomes over the general public [127]. Another study discusses an increased incidence of brain tumors and non-Hodgkin lymphoma in those with CFS/ME, including analyses of two outbreaks of fatigue-related illnesses and the frequency of cancers in those involved. However, the authors of this report state that further research is needed because a causative factor for carcinogenesis and manifestation of fatigue could not be determined [128]. Another study found an increased incidence of brain tumors in CFS/ME patients, but not non-Hodgkin Lymphoma (NHL) [129]. Moreover, there are subgroups of cancer therapy patients where severe fatigue may be connected to CFS/ME [130]. A recent study indicated that CFS/ME is associated with an increased risk of various subtypes of NHL [131] which was found in another study mentioned previously. The same study also found that CFS/ME was associated with cancers of the pancreas, breast, oral cavity, and pharynx, although not after correcting for the multiple comparisons used in statistical analysis. One comparative study looked at prostate cancer patients and CFS/ME patients for evidence of common biomarkers. The authors found that both prostate cancer patients and CFS/ME patients showed modulation of the expression of P2Rx7, a metabolite-detecting transmembrane receptor, and HSPA2, a heat shock protein implicated in a wide range of cellular processes, compared to controls. Expression of DBI, a GABA-A receptor modulator, was also correlated with the severity of fatigue in both prostate cancer fatigue (PCF) and CFS/ME patients [132]. Another study found differences in the “psychophysiology” of patients with cancer-related fatigue and CFS/ME. They found that patients with cancer-related fatigue showed higher hs-CRP levels, an inflammatory marker, and reduced HRV-index scores, a measure of heart rate variability, compared to the CFS/ME group, and that the CFS/ME group could be distinguished by EEG. However, this study did not include a healthy control group [120]. These reports present some evidence that CFS/ME could be connected to CRF, or cancer in general, in at least a subset of cases, however further studies are required to establish a conclusive link. 

There are a variety of proposed mechanisms that could underlie the etiology of CFS/ME and CRF. In the following section, we describe several biological processes that have been putatively implicated in both CRF and CFS/ME. Given the heterogeneous nature of both diseases, it is very likely that the following list is not an exhaustive one. However, there is some evidence in the literature that suggests that at least a subset of CFS/ME and CRF patients exhibit signs of the following biological phenomena. These processes could conceivably promote the initial onset of each syndrome or participate in the maintenance of the pathophysiological state. Among nearly all the articles that were reviewed for the present report, a common theme was the idea that CFS/ME is a multifactorial disease that involves some combination of normal processes going awry. As we discuss in the following section, many of these processes are likely to be interlinked in the context of each disease. 

### 3.1. HPA-Axis Dysfunction

Dysfunction of the hypothalamus-pituitary-adrenal (HPA) axis has been suggested as a contributing factor in the underlying etiology of both CFS/ME and CRF. Currently, there is controversy concerning whether the HPA axis plays a role in the genesis of fatigue symptoms in general [133]. One study reported detectable dysfunction in CFS/ME patients; the authors proposed that administration of cortisol at physiologic levels may be tried as a putative therapy for CFS/ME where there is evidence of adrenal dysfunction [134]. Another report corroborated these findings and discussed that low cortisol levels are more likely to be present in women than in men, that a multidimensional etiological model for CFS/ME is likely, that cognitive-behavioral therapy may be useful in addressing the deficiency in cortisol levels, and finally that further research is needed to fully understand the involvement of HPA axis dysfunction in CFS/ME [135]. Several other reports discussed this feature of CFS/ME, connections to genetic changes, inflammatory responses, and neuroendocrine changes, and similarly suggested that further research is needed to elucidate these connections [136,137,138,139]. 

In terms of CRF, the involvement of HPA axis dysfunction also remains somewhat contentious. One review of the literature suggested that multiple factors likely contribute to CRF, with correlated symptoms of depression, anxiety, and chronic pain [140,141,142]; some of these symptoms also fall in the diagnostic criteria of CFS/ME. Additional factors were also identified, such as comorbid medical conditions and different distribution depending on demographic factors [142,143]. Numerous studies on a myriad of biological parameters that could potentially induce fatigue—including hemoglobin, albumin, and thyroid hormone levels—have been mostly fruitless in explaining the fatigue syndrome in cancer patients [144]. This could also reflect a heterogenous etiology in CRF, as we suspect the same for CFS/ME. The authors note that treatment with pro-inflammatory cytokines, such as interleukin-8, interleukin-6, and tumor necrosis factor-alpha, at physiologic doses, appears to facilitate HPA axis dysfunction and promote fatigue-like symptoms. As with CFS/ME, further research is needed to establish a causal relationship between CRF and HPA axis dysfunction.

### 3.2. Serotonin

Another study noted similarities between CFS/ME and cancer-related fatigue and described some proposed mechanisms [145]. The author noted that 5-HT metabolism and neurotransmission could potentially be implicated in both CFS/ME and CRF, as another study found increased free tryptophan in the blood of CFS/ME patients [146]. While some investigators showed that administration of 5-HT or selective serotonin reuptake inhibitors reduced the capacity for exercise in humans, others investigating CRF in specific cancers found no connection [147,148,149]. In some types of lung cancer, the metabolism of tryptophan to kynurenine may result in further conversion to neurotoxic metabolites that are associated with fatigue [150,151,152]. Currently, the link between CRF and 5-HT metabolism remains a controversial but very interesting area of ongoing research. Another study examining a chronic fatigue model in rats found that the quantity of serotonin increased following exercise and this was associated with the induction of chronic fatigue [153].

It is very interesting to us that serotonin may be involved in CFS/ME and CRF. If a common link is found, it may explain the underlying mechanisms behind the disease in at least some of the affected populations. We are further interested in the potential connection these two diseases may have to low-dose radiation exposure, as serotonin is known to be involved in radiation-induced bystander effects as well. The involvement of serotonin in radiation-induced bystander effects has been extensively documented in the literature by our group and others [120,127,154,155,156,157]. Serotonin was demonstrated to be required for bystander effects in one study [156], and this was proposed as a potential explanation for the inter-laboratory variation in bystander effects; this was concluded because the commercially available fetal calf serum batches that were used in cell culture were found to contain variable quantities of serotonin. Another group found that there appeared to be an interaction between serum serotonin levels and p53 status with respect to cells’ ability to respond to bystander effects [158]. Supplementing serotonin in culture medium produced “a modest but significant increase in (micronuclei) formation”, while low serotonin levels again abrogated bystander effects in the p53 wild-type cell line. However, the addition of serotonin to the p53-null cell line appeared to allow the cells to respond to bystander signals. 

These findings are very interesting and should be investigated further. We surmise that low-level stressors—low doses (<100 milligray or mGy) of ionizing radiation, for example—may instigate fatigue symptoms in those with some underlying and predisposing factors. This could be diagnosed as CFS/ME, however, depending on the context of symptom onset, may be diagnosed as CRF. Predisposing factors could include bacterial or viral infection, genetic background, or serotonin imbalance. There may be some connection between excess serotonin, a permissive environment for the reduction of oxidative metabolism, radiation exposure, and bystander effects. These are excellent avenues of research for prospective epidemiological studies, and hopefully, future research will be able to tease out the connection between these phenomena.

### 3.3. The Circadian Clock

It should first be noted that the suprachiasmatic nucleus in the brain controls the mammalian circadian clock, and some studies have found that serotonin is a direct regulator of the phase of this clock. Recent studies have also suggested that serotonin is involved in regulating the circadian clock in mammals by directly modulating the expression of specific genes in the brain [159]. The circadian clock is also regulated by external, environmental factors, such as day and night cycles. 

There are a few studies that investigate a link between the circadian clock and CFS/ME. The rationale behind these studies is that CFS/ME presents with symptoms that are associated with trouble falling, staying asleep, or otherwise unrestful sleep. Insomnia is comorbid with a panel of mental health disorders, including anxiety, depression, and substance abuse disorders [160]. A systematic review of the literature by Shan et al. [161] found that reduced serotonin transporters were found in several studies examining CNS abnormalities in CFS/ME patients. One study examining eight patients with CFS/ME concluded that the patients exhibited lower daytime activity and less regular activity-rest cycles due to the illness, noting that “some of the symptoms of chronic fatigue syndrome (CFS) are the same as for disrupted circadian rhythm” [162].

Alterations to circadian function are present in some patients with cancer; associated changes include disruptions to endocrine rhythms, metabolic processes, and the immune system [122,163,164,165,166,167,168]. Researchers have determined that cortisol levels are different between breast cancer patients experiencing fatigue and those not experiencing fatigue [169]. As in CFS/ME, sleep disturbances and disorders are common in patients with cancer [170,171]. Several groups reported that fatigue in cancer patients is associated with altered activity-rest activity patterns [124,167,172]. The causes of these disturbances in cancer patients are not fully understood. However, it is suspected that a combination of genetic, psychosocial, environmental, behavioral, and metabolic causes are to blame [122]. It is also likely that tumors have a direct effect on the regulation of host rest/wake cycles through the tumor microenvironment and influence immune responses [122]. This provides a connection to potential neuroimmune involvement. Changes in cortisol levels can change the function of immune cells, which in turn promotes proinflammatory cytokine production [122,173,174]. Moreover, altered immune function is coincident with flattened cortisol rhythms in patients with breast cancer [122,175,176].

### 3.4. Inflammation and Immunity

CFS/ME has been observed to develop the following infection and has been reported following cases of Epstein-Barr viral infection [177]. Interestingly, a report published in the early nineties demonstrated the presence of retroviral sequences related to T-lymphotropic virus type II in CFS/ME patients [177]. These viruses can cause a specific type of cancer in humans known as adult T-cell lymphoma/leukemia. Additionally, they can cause a demyelinating disease. Infection with HTLV-2 is associated with neurological abnormalities, including sensory neuropathies, abnormal gait, cognitive impairment, and erectile dysfunction [178,179,180]. Even though these studies can still be found in the literature, several groups could not replicate these findings; therefore, this hypothesis is no longer supported [181,182,183]. Another recent review notes the immunological similarities between CFS/ME and cancer and proposed common links to fatigue symptoms [184].

A review by Noda et al. [185] reported that CFS/ME is a disease that may be linked to neuroinflammation, which is discussed in further detail above. One study found that decreased natural killer cell activity was positively correlated with the severity of CFS/ME [186]. Several studies have examined the role of cytokines in CFS/ME, which is also described above. One report noted that the cytokine profile in the plasma of female CFS/ME patients appeared to be consistent with “processes active in latent viral infection” [187]. Another paper by Broderick et al. [188] showed significant differences in IL-8 and IL-23 concentrations in adolescents with post-infection CFS/ME. However, a critical review of the methods used in the literature cautioned that the potential variance in cytokine responses between individuals will make it difficult for future studies to replicate findings; we argue that this also speaks to the heterogeneity of the disease at a “systemic level”, and that this probably corresponds to heterogeneous responses at the molecular level as well. 

It is known that oncogenesis and cancer therapies—including surgery, chemotherapy, radiotherapy, and targeted therapy—are associated with increased levels of plasma cytokines and inflammation [189,190,191,192,193]. Some proinflammatory cytokines have also been implicated in CRF. In particular, TNF-alpha and IL-1beta are implicated in many mechanisms believed to be responsible for fatigue. Supplementation of TNF-alpha is known to provoke behavioral changes, including lethargy, and when used in immunotherapy, a common side effect is fatigue [194,195]. Therefore, it is suspected that cytokine signaling networks coordinate biological responses following exposure to some injurious, cytotoxic factor—such as ionizing radiation chemotherapy, et cetera—resulting in inflammation and cytokine signaling.

With respect to ionizing radiation exposure, cytokines have been shown to be involved in bystander signaling as well. An analysis of cytokine secretions was found to be cell line-specific in one study, with a smaller increase in overall concentration after fractionated rather than acute doses [196]. The highest level of cytokine secretion was also associated with the poorest survival in A549, a human adenocarcinoma cell line. In glioblastoma cells, gamma irradiation caused the release of IL-8 and IL-6, resulting in bystander effects in reporter cells [197]. Interleukin-8 (IL-8) is released into extracellular space following irradiation of T98G cells in vitro in a time-dependent and dose-independent manner [198]. IL-6 is also known to be released from irradiated cells and has been implicated in bystander responses [199]. These changes to inflammatory responses are expected with ionizing radiation exposure, however, it is noteworthy that these responses do not exhibit a clear dose-dependent relationship.

### 3.5. Metabolic Changes and Nutrient Deficiency

Since the recognition of CFS/ME as a debilitating disease, researchers have suspected that the symptomatology of the disease does not simply result from psychological or neurological causes. It was believed that a lack of energy to perform routine tasks, which were previously performed without difficulties, could stem from a lack of energy in skeletal muscle or the tissues supporting it. Researchers suspected that ATP deficiencies could account for the lack of this energy. An analysis of blood samples from 74 CFS/ME patients found significant evidence of mitochondrial dysfunction, and specifically deficiencies in ATP production. A report by Naviaux et al. [22] found that CFS/ME appears to be “a highly concerted hypometabolic response to environmental stress that traces to mitochondria”. The researchers utilized broad-spectrum metabolomics and assessed 84 CFS/ME patients; 612 metabolites in plasma were analyzed that were involved in 63 biochemical pathways. Patients showed significant abnormalities in approximately one-third of the metabolites examined. Abnormalities were related to branched amino acid synthesis, phospholipid metabolism, cholesterol metabolism, and mitochondrial metabolism. The researchers demonstrated a relatively homogenous metabolic response in the cohort that was examined. Another report using the metabolic profiling of blood and urine samples from CFS/ME patients showed elevated blood glucose and reduced blood lactate, urine pyruvate, and urine alanine [200]. The researchers stated that the dysfunction of glycolysis could explain these results, with fewer metabolites available for reactions in the citric acid cycle. Additionally, the researchers presented evidence for oxidative stress in these patients. However, another report found normal lactate metabolism and oxygen uptake in CFS/ME patients [201]. Another study showed abnormalities in fatty acid and lipid metabolism. A systematic review of the literature by Shan et al. [161] found that regional hypometabolism was observed by several groups of researchers. These are just a few of the studies that have examined metabolic changes in CFS/ME patients. Much of the current research focuses on these potential biomarkers and explains discrepancies in results between different research groups [202].

We have discussed previously that metabolic changes are a hallmark of carcinogenesis. Further, the presence of a tumor microenvironment promotes these changes even in the presence of oxygen. CRF has been hypothesized to result from changes to energy demands in the body following the development of a tumor. It may be anticipated that a tumor will use a considerable portion of available nutrients to accumulate biomass, thereby leading to metabolic deficiencies in the patient. This results in a multitude of symptoms, however, an energy deficit may be explained partly through a lack of metabolites or other reorganization of the metabolism at the molecular level. 

Alteration of ATP production is known to be involved in responses to ionizing radiation as well [61,101]. We suspect that exposure to low doses of ionizing radiation could act as a causative factor for the formation of CFS/ME in some cases. While there is a dearth of research on the ability of bystander effects to promote oncogenesis, it is known that radiation responses can create conditions that are conducive to the formation of cancer. For example, the release of ROS promotes an oncogenic state because it leads to the damage of healthy tissue, and at the molecular level, oxidative or reductive damage to DNA; this is also known to result in genomic instability, in theory promoting carcinogenesis [114,203,204,205,206]. In CFS/ME or CRF, attenuation of oxidative metabolism by bystander effects [61,101] after exposure to ionizing radiation or another environmental stressor could cause fatigue symptoms in theory. This is a very exciting area of research that may be promising in elucidating the etiology of both diseases.

In the last section, we reviewed what mechanisms may be behind the etiologies of CRF and CFS/ME. Because there is considerable overlap between the symptoms, there is reason to suspect similar underlying mechanisms. Furthermore, there is considerable evidence of this in the literature; however, more work needs to be done before it can be concluded that there is a connection.

Cancer-related fatigue and associated symptoms are often present in cancer patients—even those not undergoing treatment—however, the underlying pathophysiology is not well understood. We discussed several mechanisms that could be behind these symptoms, including increased cytokines, dysregulation of the HPA axis in the brain, disruption of sleep and circadian rhythms, muscle loss, nutrient deficiency, and genetic changes. Fatigue can sometimes be associated with a separate condition like anemia due to cancer, however, the fatigue is usually idiopathic. Additionally, many patients who recover from cancer continue to report fatigue long after remission, indicating that these changes may become chronic in some cases. On a cellular level, it may be that these changes are transferred to cell progeny, either through inheritance or maintenance of some tissue microenvironment. 

It is widely appreciated that CFS/ME and cancer are very different diseases. However, we believe that they may have similar underlying etiologies in some cases. Cancer is known to promote drastic changes to cellular metabolism, which causes proliferation and growth under anaerobic conditions. It is suspected, based on numerous previously published articles, that CFS/ME also produces changes to cellular metabolism and may cause the downregulation of energy production. We hypothesize that CFS/ME and cancer may be related conditions that lie on a spectrum of possible biological effects, with a general reduction of metabolism and growth in CFS/ME and promoted anaerobic growth in cancer. The underlying causative agent(s) for the instance of cancer and CFS/ME may dictate this response. Given this consideration, the development of a neoplasm versus maintenance of a fatigued state could depend on genetic background, nutritional availability and microenvironment, presence of other toxins, or even the dose of the injurious agent. These toxins could include heavy metals or radioisotopes, for example. Alternatively, the response may be dependent on the individual, with previous exposures “tempering” or sensitizing organs and tissues to additional uptake. A potential example of something that could cause the initiation of SIPI (stress-induced phenotype instability) is exposure to very low doses of ionizing radiation. When a person is exposed to one of these stressors, multiple outcomes are possible, and different dose relationships may be inferred. For example, hormetic responses, a biphasic response, tolerance, or hypersensitivity. It will likely be very difficult to determine how cancer fatigue and CFS/ME are linked unless additional research is conducted. Furthermore, it is important to note that this hypothesis is yet to be proven or refuted.

As previously discussed, there are many different types of cancer, each with potentially unique underlying mechanisms implicated in their manifestation. In terms of CRF, it is known that this type of fatigue affects many different cancer patients. So far, it is unclear which type of cancers could be connected to CFS/ME and this connection has yet to be proven. It is suspected that the modified energy metabolism in cancer can be connected to energy deficiencies in CFS/ME, however, it is again not yet known which type of cancer could be connected to the disease and what mechanisms could account for fatigue symptoms. We summarize both conditions in Table 1, providing an overview of symptoms, possible causes, and possible underlying mechanisms. 

In another review paper, Rusin et al. [36], discussed some potential future directions for research after hypothesizing that CFS/ME may be traced to mitochondrial insufficiency. We included a summary of these ideas in the following section and adapted them to better investigate the possible links to CRF and cancer.

## 4. Possible Implications to Clinical Practice in the Management of Fatigue

If a connection between CRF and CFS/ME is established, the context under which the fatigue symptoms emerge is important for diagnosis and determining treatment. For example, in the case of a cancer patient reporting fatigue symptoms, it could be initially assumed that cancer itself is the primary cause, however, this is not necessarily the case —as described previously—if other possible causes are present. If the fatigue is debilitating and interferes with daily activities, then counseling and basic education are usually prescribed as a first step [217]. Following this, the fatigue can be assessed in depth by an examination of associated systems, and then several treatable causes must be discounted; these include pain related to cancer, emotional distress possibly related to cancer, anemia, cancer-related sleep disturbances, nutritional deficiencies, comorbidities, and a possible link to a treatment regime. Assuming none of these causes can be identified, then the best course of action may be symptom management. 

It is proposed that, whether or not CRF and CFS/ME have an underlying cause or set of causes, that CFS/ME management should be approached in a similar fashion in clinical practice. First, a diagnosis of CFS/ME should be made based on excluding other potential criteria that may cause fatigue [218]. It is therefore important to have a complete medical history of the patient and perform a complete physical examination. As previously described, fatigue may be caused by another medical condition, emotional distress, sleep disturbances, and nutritional deficiencies; therefore, the exclusion of these factors is important in determining an appropriate approach. To further complicate matters, the CFS/ME condition is oftentimes associated with sleep disturbances [170], so teasing out the likely cause of these disturbances would be very useful for the management of the symptoms. In adolescents, cognitive behavioral therapy appears to be helpful in a majority of cases, so this could be indicated depending on the underlying cause [218]. In cases where this approach is not helpful, then the possibility of a metabolic or other physiological cause may be evidenced. Because the research on possible biomedical markers is still highly controversial in the literature, it is difficult to recommend specific assays to be used in diagnosis, as more research and evidence are required. Future research efforts should focus on standardizing methods for metabolomic screening of CFS/ME such that findings can be better compared between studies in the future [219].

## 5. Conclusions

In CFS/ME and CRF, there is significant overlap in terms of symptoms and presentation. Both syndromes could conceivably result from exposure to some stressor, such as ionizing radiation, and there appears to be evidence in the literature that not only supports this but also the notion that CFS/ME and CRF may be connected etiologically. Neurological dysfunction, changes in serotonin, the circadian clock, elevated inflammation, metabolic deficiencies, and genetic changes are candidates for mechanisms in both diseases, although further research is still needed. At present, the effect of radiation dose on the induction of fatigue and these conditions is still unclear, and further research is needed to link radiation to the two diseases—particularly CFS. Considering that both CFS/ME and cancer are very heterogenous and diverse conditions, we suspect that a combination of these factors may be present in CFS/ME patients and those suffering from CRF. Identification of common biomarkers in patients suffering from one disease or another is required, as this may determine where effective therapy can be applied.

## Figures and Tables

**Table 1:** A summary table comparing CFS/ME and cancer-related fatigue. Some symptoms and relative frequency are listed. Possible causative factors are also listed and are appraised on the amount of evidence in the current literature. Finally, possible biological processes are listed for both diseases, along with how much support appears to be present in the present literature. Entries marked with an asterisk continue to be contentious topics in the literature.

	CFS/ME	Cancer-Related Fatigue
Symptomatology		
Fatigue	Very common [3]	Very common [121]
Post-exertional Malaise	Very common [15]	Uncommon [120,122]
Problems Concentrating	Very common [3]	Very common [140]
Reduced Physical Activity	Very common [23]	Very common [207]
Sleep Disturbances	Very common [23]	Very common [122,171]
Emotional Problems	Very common [23]	Common [130,169]
Physical pain	Common [23]	Very common [192]
**Possible Causative Factors**		
Toxins/Cytotoxins/Mutagens	Some evidence [95,208]	Considerable evidence for carcinogenesis [93]
Radiation	Little evidence [33,35]	Considerable evidence for carcinogenesis [209]
Viral/bacterial infection	Some evidence [170,181,210]	Considerable evidence for carcinogenesis [69,211]
**Possible processes and degree of evidence in the literature**		
Metabolic dysfunction	Some evidence * [15,22]	Some evidence [122]
Endocrine dysfunction	Some evidence * [133,135,212]	Considerable evidence [122,171]
Immune dysfunction	Some evidence * [14,170,186,213]	Considerable evidence [144,214]
Neurological dysfunction	Considerable evidence [215,216]	Some evidence [122]

## Data Availability

Not applicable.

## Supplementary Materials

The following supporting information can be downloaded at: www.mdpi.com/article/10.3390/ijms23020691/s1.
