# Supplementary material for: Commonalities in the Features of Cancer and Chronic Fatigue Syndrome (CFS): Evidence for Stress-Induced Phenotype Instability?
Source: Int J Mol Sci. 2022 Jan 8;23(2):691. doi: 10.3390/ijms23020691 (PMC8775947; doi:10.3390/ijms23020691)
Supplement: Supplementary file 1 [file ijms-23-00691-s001.zip › ijms-1509462-supplementary.pdf]

ARTICLE	DISEASE/PROCESS	SYNOPSIS/KEY FINDINGS
Servaes et al. 2001	CFS/CRF	-study examining CFS and CRF patients<br>-19% of disease-free cancer patients are fatigued<br>-no relationship between fatigue and type of cancer<br>-fatigue associated with lower motivation, concentration, physical activity<br>-relation between fatigue, depression, and anxiety<br>-similar "fatigue experience" between CRF and CFS
Stussman et al. 2020	CFS	-study examining CFS patients<br>-range of symptoms attributed to exertion<br>-core symptoms: exhaustion, cognitive difficulties, neuromuscular complaints<br>-unique variations particular to different individuals<br>-post-exertional malaise varies greatly between individuals and causes a reduced quality of life
Park et al. 2019	CFS/CRF	-study examining CFS and CRF patients<br>-both CFS/CRF patients show comparable levels of fatigue but different presentation<br>-CRF patients had higher high sensitivity C-reactive protein (hs-CRP) levels and reduced heart rate variability (HRV)-index<br>-different pathophysiological mechanisms underlie CFS and CRF; inflammatory markers and EEG markers may distinguish the diseases
Franc et al. 2014	CRF	-fatigue affects many with cancer<br>-often diagnosed during treatment<br>-oftentimes the feeling is specific to the individual and may be subjective<br>-pathogenesis is unknown still<br>-CRF causes reduced quality of life<br>-treatment may be attempted based on the suspected underlying cause
Ryan et al. 2007	CRF	-review about the suspected mechanisms of cancer-related fatigue<br>-very prevalent in cancer patients with a large effect on quality of life<br>-still poorly understood and may involve contributions from comorbid disorders, therapy, or cancer itself<br>-suspected to often involve dysregulation of several physiological and biochemical systems<br>-may include dysregulation of several systems: HPA axis, 5-HT, ATP metabolism, circadian rhythms, cytokine dysregulation, and muscle alterations<br>-however, still very little evidence directly linking these processes to CRF
Saligan et al. 2015	CRF	-systematic review paper of current literature on cancer-related fatigue

		-47 current articles reviewed; 25 cross-sectional and 22 longitudinal<br>-findings suggest that CRF is liked to immune/inflammatory, genetic, neuroendocrine, and metabolic markers<br>-gaps exist in current research
Berger et al. 1998	CRF	-study of CRF in breast cancer patients, specifically related to adjuvant therapy (chemotherapy)<br>-the study found unexpected variance in reported fatigue over the course of the treatment cycle, peaking during treatment and waning at midpoints
Jereczek-Fossa et al. 2002	CRF	-radiotherapy-induced fatigue is very common in patients undergoing radiation therapy<br>-poorly understood etiology<br>-may depend on site of tumor and treatment regime<br>-psychological mechanisms have been preposed<br>-some potential treatments include group psychotherapy, relaxation therapy, exercise, and sleep
Kamal et al. 2019	CRF	-a study using dosimetric data from disease-free nasopharyngeal cancer patients<br>-dosimetric data obtained from several regions in the brains following radiation therapy<br>-a “dose-fatigue” relationship is present for the pituitary gland, determined through patient and observer reporting
McManimen et al. 2016	CFS	-study to determine if CFS patients die earlier than the rest of the population from the cause—56 cases were examined<br>-significantly increased risk of earlier all-cause death, cardiovascular-related mortality<br>-directionally lower age of death for suicide and cancer<br>-findings should be replicated due to sample size of the study and the use of only severely ill patients
Levine et al. 2011	CFS, Cancer	-attempt to use population-based data to determine relationship between cancer and CFS, if any<br>-some evidence of increased non-Hodgkin lymphoma (NHL). however this needs follow-up studies<br>-not all CFS patients exhibit immune dysregulation, and a single causative agent is highly unlikely between both diseases<br>-focusing on subgroups in each disease may prove more useful
Levine et al. 1994	CFS, Cancer	-CFS has been associated with immune dysregulation in the past, however this is very contentious in the literature

		-it is difficult to warrant association of CFS with cancer given the then-current data<br>-no consistent trends associating NHL with CFS<br>-upward trend in the incidence of CNS tumors may be related to a general increase in cases in the general population<br>-several recommendations given for later studies, including latent period for specific tumors
Servaes et al. 2002	CFS/CRF	-study of 57 severely fatigued but disease-free breast cancer patients and in 57 gender and age matched patients with CFS<br>-CFS patients tend to self-report worse level of fatigue, functional impairment, physical pain, pain, and efficacy<br>-a subgroup of severely fatigued breast cancer patients scored equally to the CFS patients<br>-some similarities between the patient groups but also a great deal of differences—cognitive-behavioural therapy should differ between the patient groups
Chang et al. 2012	CFS/Cancer	-epidemiological study on 1.2 cancer cases<br>-sought to determine whether CFS can be linked to cancer based on the hypothesis of immune dysfunction and metabolic dysregulation<br>-CFS seems to be associated with some types of NHL<br>-these numbers remained elevated after excluding patients with autoimmune conditions or other conditions unrelated to NHL or CFS<br>-some associations with other cancers, however significant after multiple comparisons
Light et al. 2013	CFS/CRF	-prostate cancer patients undergoing androgen-deprivation therapy with self-reported fatigue (PCF) and CFS patients were assessed for several potential biomarkers using RT-qPCR<br>-PCF patients showed high expression than CFS patients of two genes involved in immune transcription (NR3C1 and TLR4), chemokine CXCR4, and SOD2 (superoxide dismutase)<br>-CFS patients showed high metabolite detecting receptors (P2Rx7) and lower HSPA2<br>-both CFS and PCR patients showed correlations with fatigue severity that were similar between the groups in a GABA-A receptor modulator
Cleare 2004	CFS	-many studies have reported CFS may be linked to alterations in the HPA axis<br>-early prospective studies may indicate no changes in the early stages of the illness

		-changes may be reversed by modifying behaviour<br>-this article presents the opinion that there is no actual change in the HPA axis of CFS patients that causes the disease, but that the etiology is multifactorial and play a role in “exacerbating or perpetuating symptoms late on in the course of the illness”
Holtorf 2011	CFS	-there is significant controversy regarding the relevance of HPA-axis dysfunction in CFS<br>-some studies exist that have demonstrated a deficiency through stimulation tests<br>-CFS dysfunction of the HPA axis is in the pituitary-hypothalamic level mostly according to studies<br>-treatment of low physiologic doses of cortisol have been shown to be helpful in these cases, so administration of cortisol (<15 mg) may be useful in treatment
Papadopoulos & Cleare 2012	CFS	-there is considerable evidence of reduction of cortisol levels in some CFS patients, and these changes appear more pronounced in women than in men<br>-the hypothalamic–pituitary–adrenal (HPA) axis may be to blame for this, along "with attenuated diurnal variation, enhanced negative feedback and blunted response to challenges"<br>-ow cortisol levels may contribute to symptoms and an overall worsened outcome<br>-multidimensional etiological model is most probable<br>-low cortisol appears late in the illness and may be moderated by exercise, existing mental health conditions such as depression, early trauma, and psychotropics<br>-cortisol levels may be improved by cognitive behavioral therapy<br>-better research design is required for future studies on CFS
Vangeel et a. 2015	CFS	-study quantified DNA methylation in the 1F promoter region of NR3C1, a biomarker for HPA-dysfunction<br>-overall methylation was lower in CFS patients compared to controls<br>-no significant differences between patients with childhood trauma and those without trauma<br>-results consistent with HPA-axis dysfunction in CFS patients
Houdenhove et al. 2009	CFS	-narrative review<br>-the etiology of CFS is poorly understood<br>-although HPA axis dysfunction is common in CFS patients, it is unclear is it is causative or merely a consequence of the illness<br>-HPA axis dysfunction is hypothesized to point to “neurobiological stress”, imbalance between

		glucocorticoid and inflammatory signalling pathways, and a "cytokine-induced sickness"<br>-these processes are hypothesized to underly CFS where HPA-axis dysfunction in present
Van Den Eede et al. 2008	CFS	-letter to the editor<br>-there appears to be evidence for HPA-axis dysfunction in CFS, glucocorticoid negative feedback insensitivity, and blunted adrenocorticotropin response to stressors are the current and main findings
Morris et al. 2017	CFS	-systematic review<br>-considerable evidence of HPA-axis dysfunction in CFS<br>-there is also evidence that neuro-immune and pro-oxidative processes are implicated in the pathophysiology of CFS<br>-hypothesized that the above processes occur either as a consequence of HPA axis dysfunction or are causative<br>-results indicate that these are not secondary processes to HPA axis dysfunction and are most likely causative
Andrykowski et al. 2005	CRF	-study of female breast cancer patients undergoing adjuvant therapy<br>-patients completed clinical interview on measures of fatigue, distress, coping, and quality of life<br>-baseline incidence of CRF was 10%, and following treatment 26%<br>-results support multifactorial etiology—patient scores were significantly different in relation to various parameters of the questionnaire
Bower 2007	CRF	-narrative review<br>-the author considers the emerging evidence for inflammatory processes in CRF<br>-evidence suggests that this occurs both during and after treatment<br>-author identifies potential mechanisms for the inflammation, focusing on the HPA axis
Bower et al. 200	CRF	-epidemiological study on fatigue in a large sample of breast cancer survivors<br>-breast cancer patients somewhat more fatigued than the general population overall<br>-approximately one third of breast cancer survivors reported severe fatigue<br>-symptoms included increased depression, pain, and sleep disturbances<br>-depression and pain were the strongest indicators of fatigue severity

Wang 2012	CRF	-narrative review of the possible mechanisms behind CRF<br>-CRF is influenced by a number of factors, including the effect of tumor burden, cancer treatment, or other pathophysiological conditions<br>-compelling research exists in the pro-inflammatory hypothesis of CFS induction, the involvement of serotonin, anemia, and lack of adenosine triphosphate
Badawy et al. 2005	CFS	-study that assess the serotonin status of CFS patients<br>-examined various parameters of tryptophan metabolism<br>-free tryptophan was higher in CFS patients<br>-total tryptophan ratio to CAA ratio was significantly different between patient groups<br>-CAA was not significantly different between groups, however it was lower overall in men than in women<br>-there appeared to be two subtypes of CFS patients: ones with normal and high serotonin status when covaried with age end gender
Morrow et al. 2003	CRF	-it is known that fatigue and depression typically occur together in cancer patients, which suggests a common underlying mechanism<br>-cancer patients undergoing chemotherapy were assessed for fatigue<br>-if fatigue was reported, patients were placed on a paroxetine hydrochloride daily dose (a selective serotonin reuptake inhibitor) or a placebo<br>-the SSRI had no effect on the reduction of fatigue<br>-there was a mean between the groups with respect to depression, however<br>-authors hypothesize that serotonin may have no effect on fatigue levels
Barsevick et al. 2012	CRF	-systematic review of CRF and issues in the investigation of biological mechanism<br>-current research is beginning to incorporate biological and genetic perspectives of these experiences<br>-evidence suggest that the HPA axis, serotonin, circadian rhythm, and metabolism may be involved, however further research is required to elucidate these connections<br>-advances in understanding CRF will contribute to increased quality of life in patients
Roscoe et al. 2005	CRF	a study using the same SSR as Morrow et al. 2003 on breast cancer patients undergoing chemotherapy<br>-analysis showed that paroxetine managed to reduce depression effectively, however not fatigue<br>-authors hypothesize that serotonin is not the primary mechanism of CRF fatigue

O'Higgins et al. 2018	CRF	-narrative review<br>-fatigue is a debilitating symptom of cancer<br>-current research suggests that it is a multifactorial condition and likely involves immune dysfunction, muscular changes, and neuroendocrine modulation<br>-some current hypotheses are presented, including HPA axis, disruption of circadian rhythm, serotonin dysregulation<br>-authors explain different controversies surrounding these avenues of research
Dantzer et al. 2014	CRF	-narrative review<br>-the general pathophysiology of fatigue is not fully understood<br>-however, studying chronic inflammatory diseases, cancers, and neuropathologies can help elucidate these mechanisms<br>-there is convergent data from many different sources on the importance of inflammation in fatigue<br>-neurological dysfunction may also be implicated
LayVoy et al. 2016	CRF	-narrative review<br>-cancer-related fatigue is a debilitating condition, as previously reviewed<br>-authors discuss concepts such as the HPA axis, disruption of circadian rhythm, serotonin dysregulation
Liu et al. 2017	CFS	-rat model of exercise-induced fatigue<br>-study focuses on the involvement of serotonin in the manifestation of fatigue<br>-increased serotonin in the CNS is important to the adjustment to chronic fatigue<br>-serotonin plays an important role in exercise-induced fatigue
Pinho et al. 2012	RIBE	-study to determine if measurable levels of bystander factors are present in esophageal carcinoma patients' urine, and to determine if serotonin levels play a role in RIBE<br>-specifically, patients undergoing radiotherapy<br>-some of the data suggested significant effect of serotonin molecular signalling after high doses of radiation from high dose rate intraluminal brachytherapy<br>-further research required to determine if serotonin plays a role in RIBE
Lyng et al. 2012	RIBE	-study to determine the importance of serum serotonin levels in the measurement of bystander cell death

		-significant reduction in cell survival obtained in HaCaT cells treated with medium from irradiated cells, however no difference was found between bovine serum batches<br>-data suggest that serum serotonin does not play a role in the system used
Mothersill et al. 2010	RIBE	-study to again confirm or refute importance of serum serotonin levels in generations of a bystander signal from cells *in vitro*<br>-serotonin level varied widely between batches<br>-serum serotonin levels may affect ability to obtain a bystander signal and may explain different results between labs
Curtis et al. 2018	RIBE	-another study investigating the importance of serum serotonin concentration in bystander effects *in vitro*<br>-used the HaCaT cell lines and two types of HCT116 (transformed keratinocytes and intestinal epithelial cells, respectively)<br>-serotonin-depleted media significantly increased cell survival in *TP53* wild-type cells<br>-indicative of serotonin receptor heterogeneity that may underlie sensitivity to serum serotonin levels with respect to bystander responses
Kalanxhi et al. 2012	RIBE	-study investigating the role of serotonin and p53 status in RIBE<br>-medium transfer was used to assess responses in HCT116 and a breast cancer cell line, MCF-7<br>-high serotonin conditions in donor cells promoted a significant increase in bystander responses, while low serotonin levels did not show any bystander effect<br>-bystander effects can be induced in p53 null cell reporters with high enough serotonin concentrations (100 ng/mL)<br>-results indicate that serotonin levels play a role in RIBE and there could be an interaction with p53
Morin 1999	Circadian Rhythm	-narrative review paper<br>-the suprachiasmatic nucleus controls the primary circadian clock in mammals<br>-review of the circadian clock in mammals<br>-the involvement of serotonin is not immediately obvious<br>-there is conflicting information on whether serotonin affects the rhythm phase<br>-further research is ongoing
Ciarleglio et al. 2011	Circadian Rhythm	-narrative review paper<br>-the molecular biology of circadian and serotonin systems are described

		-these two systems are interconnected at the molecular level<br>-serotonin-circadian interactions in the brain may be implicated in various disorders, such as autism spectrum disorder, major depression, and seasonal affective disorder
Lee 2019	Circadian Rhythm	-narrative review paper on insomnia and how serotonin receptors may be implicated in this disorder<br>-suggested that insomnia is comorbid with several additional condition<br>-various serotonin receptors have been identified as targets for sleep aids<br>-others are potential future targets, as these have shown to be involved in sleep/wake cycles
Shan et al. 2020	CFS	-a narrative review of the neurological symptoms of CFS<br>-endothelial dysfunction is discussed and other possible mechanisms of pathogenesis<br>-other mechanisms include impaired cerebral blood flow, increased intercranial pressure, and adrenergic hyperactivity<br>-these may explain the symptoms of brain fog, headache, sleep disturbances, and hypersensitivity
Tyron et al. 2004	CFS	-study suggesting that some CFS symptoms are the same as those of a disrupted circadian rhythm<br>-CFS patients show lower daytime activity overall and less regular activity-rest cycles compared to the control<br>-the authors conclude that CFS may impair the circadian rhythm in these patients
Focan et al. 1986	CRF	-a study showing that CEA and AFP, two tumor biomarkers, can be demonstrated in individuals who do not suffer from cancer<br>-those with cancer exhibit a different circadian activity when compared to the initial group
Reinberg & Halberg 1971)	Circadian Rhythm	-various species are susceptible to chemicals that can interfere with the circadian rhythm and other biological rhythms<br>-the effects of adrenal secretion in mice induced by ATHC are discussed
Levin et al. 2005	CRF	-study examining whether those with advanced non-small cell lung cancer exhibit disrupted circadian rhythms<br>-33 patients were used in the study<br>-patients during or shortly after chemotherapy had significantly more abnormal circadian rhythm<br>-daily sleep/wake cycles are disturbed in these patients and greatly affect quality of life

Sephton & Spiegel 2003	CRF	-narrative review on the biological mechanisms that underlie the psychosocial effects on the progression of cancer, focusing on neuroendocrine and immune circadian rhythms<br>-various alterations to rhythm are discussed and potential psychosocial effects on tumor growth are also discussed<br>-review of evidence the effect of circadian rhythm dysregulation on immune responses
Mormont et al. 1996	CRF	-a study of the circadian rhythm of 30 patients with metastatic colorectal cancer<br>-a wide inter-patient variability was observed<br>-cancer patients appear to have altered rest-activity circadian rhythms<br>-further research required to determine significance to prognosis
Singh et al. 1998	CRF	-a study of 25 breast cancer patients and 15 controls, specifically on the "circadian periodicity" of various urinary compounds<br>-urinary corticoids were significantly different in breast cancer patients; these were elevated compared to controls<br>-the degree of elevation was more pronounced in advanced case<br>-mastectomy returned values closer to the control group
Bower et al. 2005	CRF	-a small study on the cortisol rhythm and fatigue patterns in breast cancer survivors<br>-fatigued survivors had a flatter cortisol slope than non-fatigued survivors<br>-results suggest "a subtle dysregulation in hypothalamic–pituitary–adrenal axis functioning in breast cancer survivors with persistent fatigue"
Evengård et al. 2005	CFS	-narrative review of CFS and suspected underlying causes<br>-causes may include immune activation, HPA axis dysfunction, and other dysfunction of other parts of the central nervous system<br>-the disease is likely to be complex and one cause likely does not exist
Roscoe et al. 2007	CRF	-narrative review on CRF<br>-fatigue is common in cancer patients<br>-symptomatology is discussed: headaches, lack of energy, malaise, disturbance of sleep, and drowsiness<br>-it may occur as a consequence of the cancer itself or possibly treatment

		-quality of life is usually affected greatly
Mormont et al. 1998	CRF	-a study of women with ovarian cancer and metastatic colorectal cancer, focusing on circadian rhythms and cortisol levels<br>-the clinical value of a 2-timepoint estimation of serum cortisol was evaluated
Petrovsky et al. 1998	Circadian Rhythms	-a study that measured IFN-gamma, TNF-alpha, IL-1, and IL12 production in 13 healthy volunteers over 24 hours<br>-cytokines exhibited distinct diurnal rhythms and peaked in the early morning<br>-pro-inflammatory cytokine rhythms may have therapeutic relevance
Kronfol et al. 1997	Circadian Rhythms	-a study that analyzed the blood samples of healthy volunteers over 24 hours<br>-the group measured ACTH, cortisol, norepinephrine, and epinephrine<br>-some biomarkers showed significant circadian rhythm, such as neutrophil percentage, CD4+ cells, CF56+ cells, number of total lymphocytes, among others<br>-most of these markers are associated closely with the regulation of cortisol
Stephon et al. 2000	CRF	-study of cortisol rhythms in those with metastatic breast cancer, with various parameters measured<br>-cortisol slope was a predictor of survival up to 7 years after measurement<br>-NK cell count was another predictor of survival<br>-metastatic breast cancer patients whose cortisol rhythms are flattened appear to exhibit earlier mortality<br>-immune biomarkers may be prognostic in metastatic breast cancer
Touitou et al. 1996	CRF	-a study of 33 breast and ovarian cancer patients and their blood serum profiles<br>-most patients had "deeply altered cortisol circadian patterns"<br>-the patients mostly exhibited erratic peaks and fluctuations, or a flattened response<br>-no relationship with tumor antigens was found<br>-these findings could be relevant to future therapies
DeFreitas et al. 1991	CFS	-a study that suggests HTLV could be involved in CFS<br>-patients were screened for the virus, and most patients were found to be positive for the antibodies<br>-twenty from a control group were not positive for the viral antigens

Kannian et al. 2010	HTLVs	-a narrative review about the human T lymphotropic viruses, outlining the different subtypes<br>-some of these viruses are associated with adult T cell leukemia and other diseases
Roucoux & Murphy 2004	HTLVs	-a narrative review about the human T lymphotropic virus subtype II<br>-the virus has been linked to a few diseases, including HTLV-associated myelopathy/tropical spastic paraparesis<br>-infection causes increased risk of arthritis, pneumonia, and bronchitis
Beilke et al. 2004	HTLVs	-a study investigating the clinical outcomes and disease progression of patients coinfected with HTLV-I and HTLV-II<br>-coinfected patients had greater neurological complications, infections, and hepatitis C, and other complications
Meeus et al. 2009	CFS/CRF	-a narrative review outlining the characteristic fatigue in CFS and cancer<br>-there is a review of several molecular factors that may underlie immune dysfunction, such as NF-kB and RNAse L<br>-the natural killer cell hypothesis is discussed in this section<br>-oxidative stress and excessive nitric oxide and their implication in both fatigue states is discussed<br>-these abnormalities may link the two conditions
Noda et al. 2018a	CFS	-a narrative review of the possible underlying causes of CFS<br>-discussion of autonomic, immune, neuroendocrine, and central nervous systems are discussed along with neuroinflammation<br>-the authors hypothesize that inflammation of microglia and astrocytes may be responsible for the immune state that leads to CFS<br>-the authors describe cytokine signaling in these cells, along with extracellular serotonin signalling
Ojo-Amaize et al. 1994	CFS	-a study conducted on blood specimens from 50 healthy individuals and 50 patients of CFS, specifically for NK cell activity screening<br>-results showed that low levels of NK activity are correlated with severity of CFS<br>-these findings may be useful especially if confirmed by other groups with larger sample sizes
Broderick et al. 2010	CFS	-a study of 16 cytokines in CFS patients and healthy controls

		-the networks in CFS patients appeared to be different compared to healthy controls<br>-there were highly attenuated immune responses in CFS according to some of these biomarkers<br>-there was also indirect evidence of diminished NK cell responsiveness to IL-12 and lymphotoxin-alpha exposure<br>-These findings may also help therapeutics and indicate IL-2, IFN-gamma, and TNF-alpha could be targets
Brodrick et a. 2012	CFS	-a small study of cytokine concentrations in plasma samples from 9 post-infection CFS patients and 12 recovered controls<br>-results showed significant differences in IL-8 and IL-23 between groups<br>-results suggest that "co-expression patterns in as few as 5 cytokines associated with Th17 function may hold promise as a tool for the diagnosis of post-infectious CFS"
Pusztai et al. 2004	CRF	-a study of 90 breast cancer patients undergoing paclitaxel chemotherapy<br>-it was suspected that changes in the blood concentration of various interleukins is associated with fatigue, flu-like symptoms, and musculoskeletal symptoms<br>-no difference in baseline cytokine levels between the groups<br>-treatment schedule-dependent changes were found with 3 cytokines: Il6, 8, and 10<br>-these also correlated with symptoms associated with treatment
Hong et al. 1995	Cytokines	-an animal study investigating whole-body and midbrain irradiation of mice and its effects on cytokines in the brain<br>-results showed that several cytokine concentrations were increased following irradiation<br>-therefore, it was concluded that these cytokines play a role in the inflammatory response upon radiation exposure of the brain
Hallahan et al. 1993	Cytokines	-a narrative review of the involvement of cytokines in radiation responses following exposure<br>-remedying some of the harmful effects of radiation therapy can be done by attenuating the production of TNF, TGF-beta, and other cytokines<br>-some cytokines are useful for cancer therapy, such as TNF-alpha which enhances killing of tumor cells<br>-more research is required to understand cytokine signalling networks

Greenberg et al. 1993	Cytokines	-a study of cytokine levels (specifically IL-1) during external beam irradiation for prostate cancer<br>-localized irradiation was found to cause increased fatigue and sleep requirement for patients<br>-changes in serum IL-1 concentrations were also noted in the patient group
Bianco et al. 1992	Cytokines	-phase I-II trial of pentoxifylline (PTX) in the downregulation of TNF-alpha production in those receiving bone marrow transplantation<br>-overall, administration of PTX resulted in reduced morbidity and mortality in patients undergoing transplantation<br>-future trials and directions are discussed, including randomized trials
Benzing et al. 1999	Cytokines	-an animal study of RGS7, a regulator of G-protein signalling, and the prevention of its degradation by TNF-alpha<br>-this process requires activation of p38<br>-TNF-alpha mediated upregulation of RGS7 may promote changes in central nervous system function upon infection of the CNS
Raison et al. 2005	Cytokines	-a narrative review of interferon alpha and its use in medical conditions<br>-adverse effects are discussed, including development of a confused state and depressive symptoms<br>-discussion of the use of antidepressants for treatment of depression induced by interferon alpha
Capuron et al. 2002	Cytokines	-another study of the major depression induced by interferon-alpha therapy<br>-studies have previously shown that this effect may be attenuated by the antidepressant paroxetine<br>-forty patients with malignant melanoma were given this antidepressant or a placebo and neuropsychiatric assessments were conducted at regular intervals<br>-several symptoms appeared including fatigue, anorexia, and pain upon IFN-alpha therapy in a large proportion of patients; later in the disease, there was a significant increase in major depression in the IFN-alpha group<br>-some symptoms, like depression, anxiety, and cognitive dysfunction were effectively treated by the drug, while fatigue and anorexia were less susceptible<br>-data indicate that different mechanisms mediate the behavioural manifestations of cytokine-induced fatigue

Desai et al. 2013	Cytokines	-a study analyzing the cytokine secretion profile of various human tumor cell lines upon gamma irradiation<br>-there was considerable variation between the cell lines in the number and magnitude of secreted factors<br>-secretion of some cytokines was found in all cell line secretions, like TNF-alpha, IL-6, and IL-8, while some were cell-line specific<br>-there are similarities and differences in the basal and radiation-induced cytokine profile of tumor cell lines and these can influence the growth and survival of bystander cells
Pasi et al. 2010	Cytokines	-a study assessing the release of IL-6 and IL-8 from gamma-irradiated human glioblastoma cells<br>-these two cytokines were found to be differentially modulated by ionizing radiation and likely coordinate the inflammatory microenvironment in the tumor
Facoetti et al. 2006	Cytokines	-a study of the gamma radiation-induced bystander effect in glioblastoma cells, specifically relating to cytokine release and receptors<br>-authors propose that cytokines could act as a radiation-induced bystander signal<br>-secreted signals may promote certain outcomes in bystander cells, such as increased cell death
Mariotti et al. 2012	Cytokines	-a study seeking to elucidate the mechanisms by which bystander transmission works *in vitro*<br>-the level of cytokine secretion from human fibroblasts was detected with an ELISA assay<br>-the effect of radiation was found to be reliant on the IL-6 pathway and shows the involvement of reactive oxygen species<br>-data also seems to suggest that increased linear energy transfer may correlate with the efficiency of radiation-induced release of cytokines
Myhill & Booth 2009	CFS	-a study investigating biomarkers of mitochondrial dysfunction in CFS patients<br>-71 CFS patients and 53 normal patients were used in the study<br>-the “ATP profile test” was used to assess the level of ATP production in neutrophils, efficiency of oxidative phosphorylation, and other biochemical markers for cellular energy production<br>-all patients except for one were found to have different combinations of metabolic lesions<br>-authors conclude that this points to the broad applicability of the ATP profile test in diagnosis of CFS

Naviaux et al. 2016	CFS	-a study of the metabolic features of CFS, which used "broad-spectrum" metabolomics of serum samples from CFS patients<br>-the data showed that the disease response was highly concerted and patients exhibited hypometabolism overall<br>-future avenues of research are discussed
Armstrong et a. 2015	CFS	-another study of the metabolomics of CFS patients<br>-researchers took 49 blood and urine samples total and these underwent spectroscopy; blood glucose was also tested along with lactate, urine pyruvate, and urine alanine—all of these indicated an inhibition of glycolysis<br>-this appears to be indicative of dysfunctional metabolism in CFS patients and increased oxidative stress
Sargent et al. 2002	CFS	-a study of CFS patients and their VO(2max)<br>-contrary to contemporaneous studies, it was found that this value did not differ between CFS and control groups when groups were age and gender matched
Germain et al. 2017	CFS	-another study of energy metabolism in CFS patients, this time focusing on fatty acid synthesis<br>-a small cohort of patients was used and the data revealed 74 metabolites which differed in their plasma signature in CFS patients compared to the control<br>-the data point to several metabolic pathways, including taurine, glycerophospholipid, primary bile acid, glyoxylate, and dicarboxylate metabolism<br>-ATP and ADP levels were also found to be affected, along with glucose and oxaloacetate<br>-potential implications for diagnosis are described
Thomas & Newton 2018	CFS	-a narrative mini-review on the various metabolic abnormalities that have been detected in CFS<br>-many studies over the years have attempted to identify biomarkers for the disease, both for diagnostic purposes and to help with determining appropriate treatments<br>-several avenues of research are discussed, including immune dysregulation, mitochondrial dysfunction, and skeletal muscle acidosis<br>-future directions for research are discussed
Martinez et al. 2010	RIBE	-a narrative review of reactive oxygen species (ROS) and their role as bystander signals<br>-review of the mechanisms of oxidative damage and paradigm shift away from targeted DNA damage

		-oxidative metabolism and regulation of inflammation are linked<br>-end of review provides potential methods that may be used for future investigations
Gorman et al. 2009	RIBE	-a study where researchers sought to elucidate the mechanisms that allow RIBE to produce genomic instability<br>-the group wanted to examine if radiation and chemotherapy can induce genomic instability and focus on mitochondrial effects and generation of ROS<br>-results demonstrate the radiation and chemotherapy bystander responses can induce genomic instability<br>-results also showed that mitochondrial function is also affected<br>-restoration of mitochondrial activity may rescue the genomic instability effect induced by RIE
